# Inferior alveolar nerve trajectory, mental foramen location and incidence of mental nerve anterior loop

**DOI:** 10.4317/medoral.21905

**Published:** 2017-08-16

**Authors:** Miguel Velasco-Torres, Miguel Padial-Molina, Gustavo Avila-Ortiz, Raúl García-Delgado, Andrés Catena, Pablo Galindo-Moreno

**Affiliations:** 1 DDS, PhD, Department of Oral Surgery and Implant Dentistry, School of Dentistry, University of Granada, Granada, Spain; 2 DDS, MS, PhD, Department of Periodontics, College of Dentistry, University of Iowa, Iowa City, USA; 3 Specialist in Dental and Maxillofacial Radiology. Private Practice. Granada, Spain; 4 PhD, Department of Experimental Psychology, School of Psychology, University of Granada, Granada, Spain

## Abstract

**Background:**

Injury of the inferior alveolar nerve (IAN) is a serious intraoperative complication that may occur during routine surgical procedures, such as dental implant placement or extraction of impacted teeth. Thus, the purpose of this study was to analyze the trajectory of the mandibular canal (MC), the location of the mental foramen (MF) and the presence and extension of an anterior loop of the mental nerve (AL).

**Study Design:**

In this cross-sectional study, a total of 348 CBCTs were analyzed. Distances from MC to the surface of the basal, medial and lateral cortical of the mandible were measured at the level of the second molar, first molar and second premolar. Location of the MF relative to the apices of the premolars, as well as incidence and anterior extent of the AL were also determined.

**Results:**

Significant and clinically relevant correlations were found between the position of the MC in women, which was located more caudal (r=-0.219, *p*=0.007; r=-0.276, *p*<0.001; right and left, respectively) and lateral (r=-0.274, p=0.001; r=-0.285, *p*<0.001; right and left, respectively), particularly at the level of the premolars. Additionally, the presence (r=-0.181, *p*=0.001; r=-0.163, *p*=0.002; right and left, respectively) and anterior extension (r=-0.180, *p*=0.009; r=-0.285, *p*=0.05; right and left, respectively) of the AL was found to be inversely correlated with the age of the patient.

**Conclusions:**

This analysis of a Caucasian population has found that the older the patient, the lower the incidence of the loop and the shorter its anterior extension.

** Key words:**Cone-beam computed tomography, mandibular nerve, mental foramen.

## Introduction

The mandibular nerve is the inferior branch of the fifth cranial nerve, also known as trigeminal nerve. Anterior to the ganglion, the trajectory of the nerve is divided into the anterior and posterior branches. The anterior branch is then divided into motor fibers, that innervate the masticatory muscles, and sensory fibers that constitute the buccal nerve. The posterior branch is also divided into motor fibers (mylohyoid nerve), that innervate the mylohyoid and anterior digastric muscles, and sensory fibers that form the auriculotemporal, lingual and inferior alveolar nerves (1).

The inferior alveolar nerve (IAN) enters the mandibular bone through the mandibular foramen (Spix spine) and runs inside the mandibular bone in the mandibular canal (MC), which ends at the mental foramen (MF), although it may present an anterior loop (AL) (1). At that location, the mental nerve emerges to innervate the inferior lip, chin and buccal gingiva. A smaller branch (i.e. the incisive nerve) continues within the mandibular bone to innervate the anterior teeth (2).

One of the most severe complications when performing intraoral surgical procedures is the injury of the IAN (3). Nerve damage can be manifested as paresthesia, disesthesia, analgesia or anesthesia (1). Depending on the duration, it can be sub-classified as reversible or permanent (if the symptoms persist for more than 6 months) (4). Although IAN injury may result from needle-induced trauma after infiltration of local anesthetic, most common clinical procedures associated with this complication are third molar extractions, implant placement, alveolar bone splitting techniques and buccal flap elevation in the premolar area, specially if the anterior loop of the mental nerve is not properly identified with the correct imaging techniques (3,5). Therefore, prior to the performance of any surgical procedures in the vicinity of the IAN, it is important to conduct a thorough evaluation, involving a meticulous radiographic examination of the MC. Although panoramic radiography may be useful, it is not completely adequate to identify the MF (6). Thus, computed tomography (CT) techniques, including cone-beam CT (CBCT), are nowadays the gold standard given the ability of performing a tridimensional assessment, reduced magnification and elimination of image overlapping (7). Noteworthy, CBCTs also reduce radiation dose in comparison with conventional CT (8).

The aim of this retrospective, cross-sectional study was to investigate the IAN trajectory through the mandibular body in the posterior area and its relationship with the posterior teeth using CBCT data from a large Caucasian population.

## Material and Methods

- Population

This cross-sectional study was reviewed and approved by the Ethics Committee for Human Research of the University of Granada (Approval number: 46/CEIH/2015). It was conducted according to the STROBE guidelines for observational studies. A random sample of 350 CBCTs obtained at the Center for Radiological Diagnosis (Granada, Spain) was retrieved and unlabeled, except for age and gender. Patients were allocated into one of three possible subgroups of edentulism (D=dentate; PE=partially edentulous; or E=completely edentulous) and sorted out by age and gender. Dentate patients were defined as those with all teeth present, excluding third molars. Partially edentulous patients were defined as those missing any tooth by hemi-arcade, excluding third molars.

- Cone-beam computed tomography (CBCT)

All samples were captured using the same equipment (Next Generation i-CAT, Imaging Sciences International Inc., Hatfield, Pennsylvania, USA) and the same settings (120 KVp, 5 mA in complete rotatory mode, a 16x8 cm field of view, with an acquisition time of 8.9 sec and 0.3 mm as voxel size). CBCT images reflecting any movement artifact, evidence of previous surgery that may hinder proper evaluation or deformities in the area of interest were excluded from the analysis.

- Radiographic measurements

A proprietary software (i-CAT Vision*) was used to obtain the different measurements in each subject on both sides (R=right; L=left). A calibrated, experienced oral and maxillofacial radiologist (MVT) performed all the measurements. All measurements were repeated twice at an interval of at least one week to minimize measurement bias. Intra-examiner reproducibility was calculated (k = 0.975).

First, all reconstructions were reformatted to position the inferior edge of the mandible horizontally. Subsequently, the following measurements were performed:

1. In the coronal plane: Distance from the mandibular canal (MC) to the lateral, medial and basal cortical at second premolar, first and second molars. In single-rooted teeth, the coronal plane was positioned along the longitudinal axis of the tooth; in teeth with multiple roots, the coronal plane was positioned at the center of the apices.

2. Location of the mental foramen (MF): the position relative to the apices of the teeth was recorded. Possibilities were: between the premolars (I4-5), distal to first premolar (D-4), apical to first premolar (A-4), mesial to second premolar (M-5), apical to second premolar (A-5), distal to second premolar (D-5) or between the second premolar and the first molar (I5-6).

3. Number of MFs per hemi-mandible.

4. Presence or absence of an anterior loop of the mental nerve (AL). If present, the distance from the most anterior location of the MF to the most anterior location of the loop was measured.

- Statistical analyses

All measurements were exported to an SPSS database (IBM SPSS Inc., v20.0, Chicago, Illinois, USA) and subsequently analyzed. Correlations were explored by Spearman’s and Pearson’s tests. Significance was established at an alpha value of 0.05. Unless otherwise noted, values were represented as a mean (SD) in mm for continuous variables and as frequency (percentage) for categorical data.

## Results

The sample analyzed consisted of a total of 348 patients, after excluding 2 due to the presence of artifacts, of which 172 (49.43%) were males and 176 (50.57%) females with a mean age of 48.57 (min-max=13-86) years. Ninety-nine were dentate (D=28.45%), 183 were partially edentulous (PE=52.59%) and 66 were completely edentulous (E=18.97%). Demographic data is summarized in  Table 1. In completely edentulous patients, AL and number of MF were the only possible measurements.

**Table 1:**
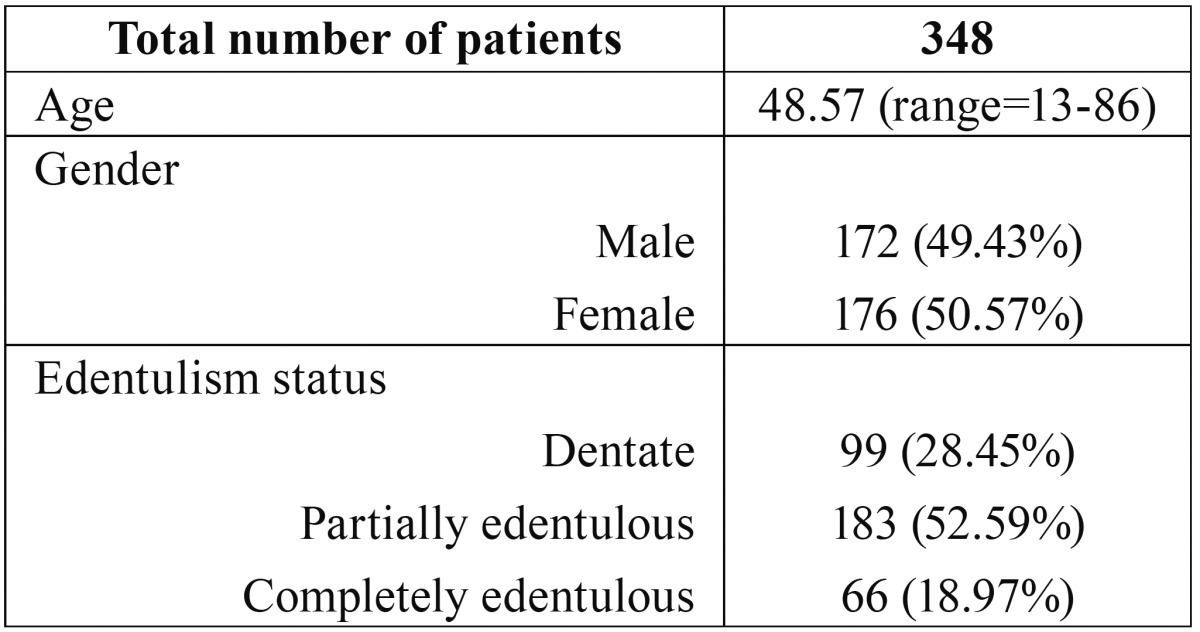
Demographic data of included subjects.

- Mandibular canal

The only significant correlations with potential clinical relevance are those related to the position of the MC at the level of the second premolar when accounting for gender. In women, the MC was closer to the basal cortical (r=-0.219, *p*=0.007; r=-0.276, *p*<0.001; right and left, respectively) and L (r=-0.274, *p*=0.001; r=-0.285, *p*<0.001; right and left, respectively).

- Mental foramen

**Figure 1:**
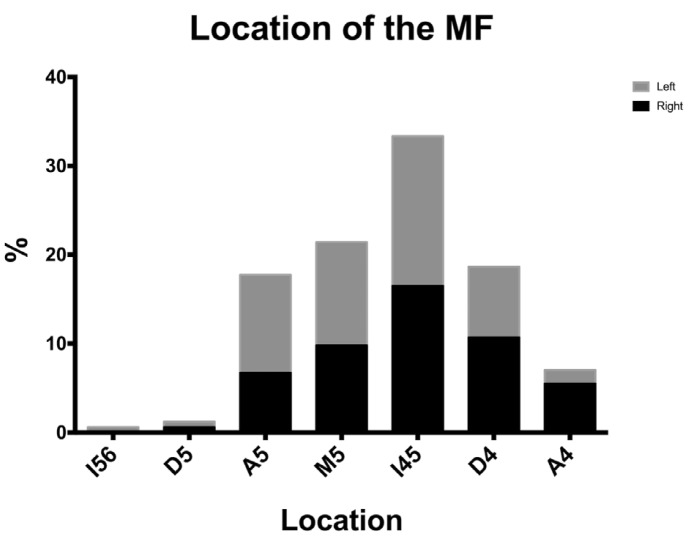
Location of the MF relative to the premolars apexes for the right and left mandible.

The most frequent location of the MF is between the first and second premolars (I4,5=33.33%), followed by mesial to the second premolar (M5=21.41%), distal to the first premolar (D4=18.65%) and apical to the second premolar (A5=17.74%), and apical to the first premolar (A4=7.03%) (Fig. 1). This means that the vast majority of subjects present their MF between the apices of the second and first premolars (98.17%). Other locations, such as distal of the second premolar (D-5=1.22%) and between the second premolar and the first molar (0.61%), were substantially less frequent.

Regarding the number of MF in each hemi-mandible, 14 subjects presented with double MF (11 right, 14 left; 3.16% and 4.02%, right and left) and only 2 subjects had triple MF (2 right, 1 left; 0.57% and 0.29%, right and left).

- Anterior loop of the mental nerve

In the sample analyzed, 60.23% and 55.94% (right and left) presented AL. The mean value of the anterior projection was 2.00 (0.98) mm and 1.92 (0.99) mm (right and left). The maximum registered projection was 6.90 mm and 7.10 mm (right and left). Differences on AL presence and morphology between males and females were not statistically significant.

**Figure 2:**
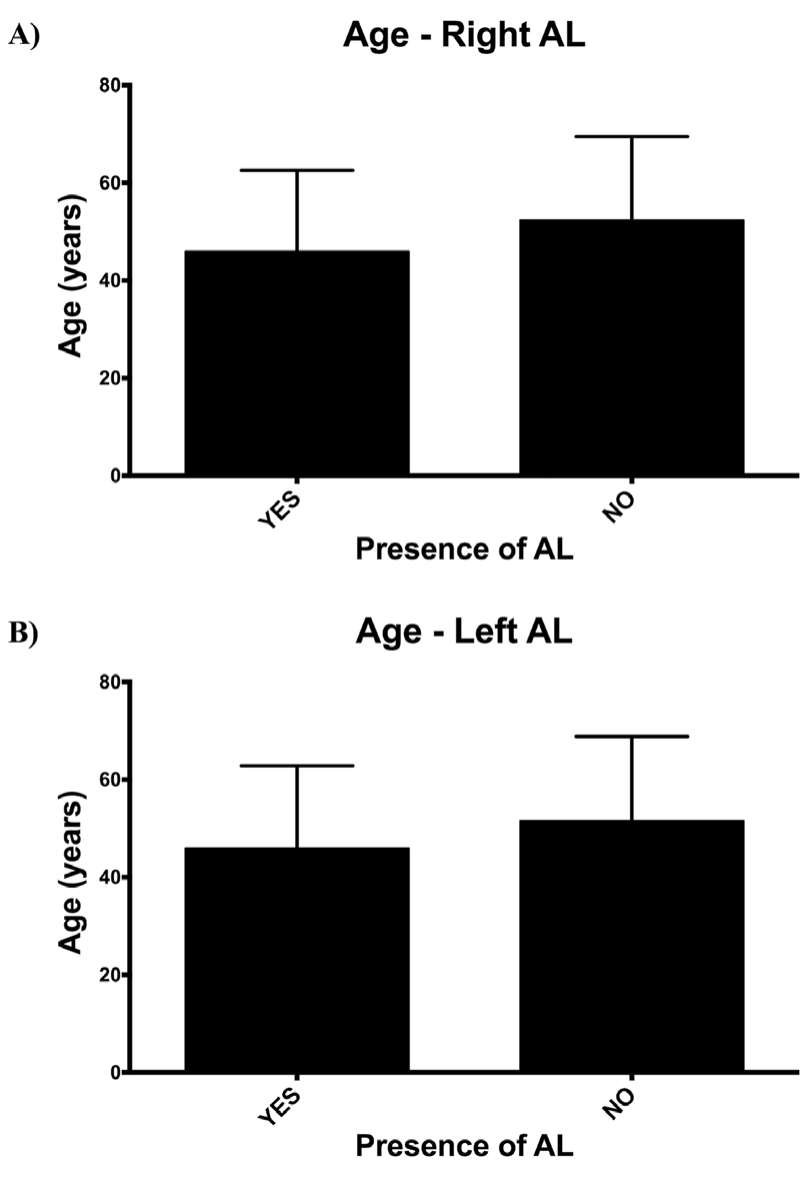
Inverse correlation between the age and the presence of anterior loop in the A) right (r=-0.181, *p*=0.001) and B) left (r=-0.163, *p*=0.002) hemimandible.

**Figure 3:**
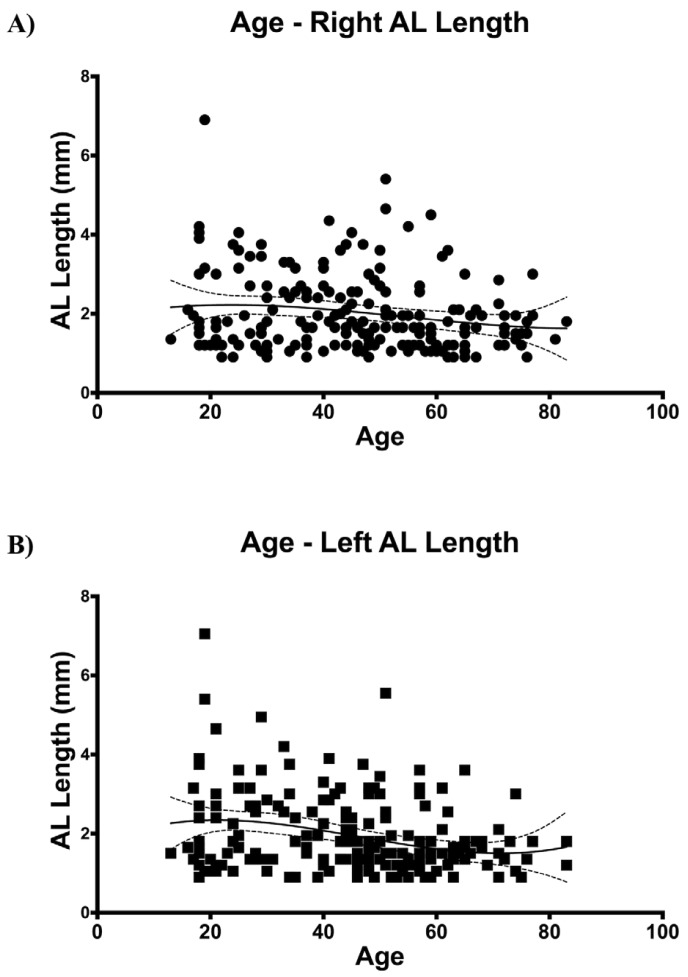
Inverse correlation between age and anterior extension of the AL for both A) right (r=-0.180, *p*=0.009) and B) left mandible (r=-0.285, *p*=0.05). Note that the third order polynomial standard curves reflect inflections at around 30 and 70 for both hemimandibles.

Age inversely correlated with the presence (r=-0.181, *p*=0.001; r=-0.163, *p*=0.002; right and left, respectively) (Fig. 2) and anterior extension (r=-0.180, *p*=0.009; r=-0.285, *p*=0.05; right and left, respectively) of the AL (Fig. 3). This means that the older the subject, the lower the incidence of the loop and the shorter its anterior extension of it.

## Discussion

Meticulous clinical and radiographic evaluation of the mandible, including the trajectory of the IAN, incisive canal and mental foramina is an essential preoperative assessment when a surgical intervention, such as tooth extraction, implant placement, periodontal surgery, etc., is planned in the mandible (9-11). Additional evaluation of other anatomical variations such as lingual con-cavities is extremely important as well (5). Panoramic radiography is likely the most commonly used diagnostic method for the localization of the mandibular canal in contemporary clinical practice given its wide availability and reduced cost. However, it is well known that lineal measurements using panoramic radiographs are not precise and not adequate to identify the MF, its diameter, shape and exit angle (6). This is mainly because of the superimposition of anatomical structures and magnifying distortions (7). On the contrary, CBCT scans have been widely regarded as the gold standard radiographic test in these and other clinical scenarios, such as locating the maxillary artery before sinus floor elevation (12). It is widely acknowledged that the information provided surpasses the risks associated with the radiation doses typically absorbed (8). Furthermore, the accuracy of CBCT for 3D analysis and linear measurements of maxillofacial bones have been confirmed by several studies (13). Assessment of anatomical structures in CBCT scans allows for the establishment of quantitative correlations between different variables and measurements, which can be used to minimize the risk of complications and to predict treatment outcomes, depending on the clinical scenario (14). One of the most severe complications that may occur when performing surgery in the mandibular bone is damage to the IAN. Thus, it is crucial to conduct a thorough pre-surgical evaluation of its trajectory, incidence and extension of the mental nerve loop and location of the mental foramen. In order to facilitate the understanding of the discussion of our findings, we have divided this section in function of those three anatomical structures.

- Mandibular canal

In this study, the distance from the mandibular canal to the basal, medial and lateral cortical bone was measured. Distance to the alveolar crest could also have been measured. However, this was ruled out since it would have been a highly variable measurement because the presence or absence of teeth would have exerted a direct effect on the position of the crestal bone. Therefore, the methodology in the current study involved analyzing the position of the MC in relation to stable anatomic structures, which are not significantly influenced by the presence or not of teeth.

In our study, average distance from the MC to the different cortical structures of the mandible were, overall, similar to those previously reported by other authors. However, our results showed higher discrepancies with Asian populations (15) than with those measured in Northern European patients (16). It is important to mention, though, that in the referred studies, smaller sample sizes were analyzed (i.e. 20 and 40 patients, respectively). Our study showed significant differences in the position of the MC at the level of the second premolar when gender was accounted for. In fact, the MC was located more apical and more laterally in women. These differences are similar to those reported in a previous study (17).

- Mental foramen

One of the most common complications affecting the mental nerve is damage at the time of flap release. Therefore, it is extremely important to locate the mental foramen. Our study analyzed the location and the most frequent anatomical variables associated with the mental foramen by using CBCT. With this technique we observed the most frequent position of the mental foramen between the first and second premolar, which correlates with previous findings conducted in Caucasian skulls (18). When considering these data altogether, the mental foramen was located somewhere between the two premolars is roughly 98% of the patients. Other locations, such as distal to the second premolar or closer to the first molar, were very infrequent (1.22% and 0.61%, respectively).

We observed that the mental foramen had one single opening in 95.97% of the cases, two foramens in 3.59% and three in only 0.43% of the population (i.e. 3/348 cases). Our findings are comparable to those previously reported in other studies (19,20). In this study, we followed the definition by Sisman *et al.* (19), in which the accessory foramen is connected to the main foramen in contrast with the nutrient foramen, that is independent.

In our sample, no correlation was found between the location of the mental foramen and the gender of the patient, although this had been previously reported (21). We did not find any other relevant anomaly, such as the recently reported bilateral absence of the foramen (22).

- Anterior loop of the mental nerve

Before performing any surgical procedure in the proximity of the mental foramen, not only its location is important, but also the trajectory of the mental nerve as it emerges from the mandibular bone. There are 3 possibilities: postero-anterior direction, at 90º o antero-posterior direction. The latter is also known as anterior loop of the mental nerve or posterior emergence, which is the most commonly found of the three (21,23). Misdiagnosis of the area could inadvertently increase the risk of damaging this portion of the nerve. It has been reported that up to 37% of the patients that receive implants in the premolar area suffer from sensitive alterations for up to 2 weeks. Of those, symptoms persist in 10 to 15%. This complication is even higher in edentulous patients with extreme atrophy of posterior alveolar segments, since there is a tendency to place the implants anterior to the mental foramen, but as close to the mental foramen as possible to avoid long prosthetic cantilevers (24).

In the population analyzed in our study, 58.08% of the subjects presented an anterior loop of the mental nerve. The average anterior projection of the loop was 1.96 (0.99) mm. This frequency and extension is similar to those previously reported, ranging from 1.50 to 2.40 mm (23–25). Other studies differ widely from those measures, either lower (26,27) or higher (18,28). Some authors even refer to this anatomical variant as an anomaly with ‘no clinical relevance’ (29), while others, in cadaveric studies, even discuss if this structure really exists or if it is just a radiographic phenomenon considering that the bony walls of the canal are not dense enough (30–33). These discrepancies are possibly due to the different methodologies and techniques being used: computed tomography, panoramic radiography, cadaver dissection or by direct access and probing of the loop of the mandibular canal. Furthermore, most of the referred studies included low sample sizes, ranging from 20 to 100, which contrasts with our study that evaluated 348 CBCTs.

Our data on presence and length of the AL did not show any differences associated to gender, in spite of what has been previously reported by other authors (24,25). We did, however, find an inverse correlation between the presence and the extension of the loop with the age of the patient. According to this, the older the patient, the lower the frequency of the loop and the shorter its extension. This could be explained by changes in the dimensions of the mental foramen. Changes in the extension of the loop with aging could be explained, hypothetically, by the continuous craniofacial growth (34). In any case, we did not analyze these aspects in particular although it deserves further investigation in future studies.

Considering these findings, it is important to consider a minimum safety distance to the AL, which may vary widely in function of the article consulted: 2 mm (17), 4 mm (26) or 6 mm (27). In practical terms, we emphasize the importance of a case-by-case evaluation.

## Conclusions

Significant and clinically relevant correlations were found between the position of the mandibular canal in women, which was located more caudal and lateral, particularly at the level of the premolars. Additionally, our study found that the older the patient, the lower the incidence of the anterior loop of the mental nerve and the shorter its extension.
